# Evidence for Finely-Regulated Asynchronous Growth of *Toxoplasma gondii* Cysts Based on Data-Driven Model Selection

**DOI:** 10.1371/journal.pcbi.1003283

**Published:** 2013-11-14

**Authors:** Adam M. Sullivan, Xiaopeng Zhao, Yasuhiro Suzuki, Eri Ochiai, Stephen Crutcher, Michael A. Gilchrist

**Affiliations:** 1 Department of Mechanical, Aerospace, and Biomedical Engineering, University of Tennessee, Knoxville, Tennessee, United States of America; 2 National Institute of Mathematical and Biological Synthesis, University of Tennessee, Knoxville, Tennessee, United States of America; 3 Department of Microbiology, Immunology, and Molecular Genetics, University of Kentucky, Lexington, Kentucky, United States of America; 4 Department of Ecology and Evolutionary Biology, University of Tennessee, Knoxville, Tennessee, United States of America; University of Zurich and Swiss Institute of Bioinformatics, Switzerland

## Abstract

*Toxoplasma gondii* establishes a chronic infection by forming cysts preferentially in the brain. This chronic infection is one of the most common parasitic infections in humans and can be reactivated to develop life-threatening toxoplasmic encephalitis in immunocompromised patients. Host-pathogen interactions during the chronic infection include growth of the cysts and their removal by both natural rupture and elimination by the immune system. Analyzing these interactions is important for understanding the pathogenesis of this common infection. We developed a differential equation framework of cyst growth and employed Akaike Information Criteria (AIC) to determine the growth and removal functions that best describe the distribution of cyst sizes measured from the brains of chronically infected mice. The AIC strongly support models in which *T. gondii* cysts grow at a constant rate such that the per capita growth rate of the parasite is inversely proportional to the number of parasites within a cyst, suggesting finely-regulated asynchronous replication of the parasites. Our analyses were also able to reject the models where cyst removal rate increases linearly or quadratically in association with increase in cyst size. The modeling and analysis framework may provide a useful tool for understanding the pathogenesis of infections with other cyst producing parasites.

## Author Summary

A large portion of people worldwide are chronically infected with *T. gondii*. Chronic infection with this parasite is characterized by formation of tissue cysts. Bradyzoites slowly replicate within cysts during the chronic stage of infection leading to a corresponding increase in cyst size. Cysts occasionally rupture and release bradyzoites that invade nearby host cells and convert into tachyzoites which can quickly proliferate. Tissue cysts can also be targeted by immune T cells and phagocytes for removal. We developed a differential equation model to investigate the cumulative effects of unknown growth and removal functions on the cyst-size distribution. We then used the AIC to select models that best fit experimental cyst size distribution data obtained from the brains of chronically infected mice. The results suggest that the within-cyst growth of bradyzoites is finely-regulated asynchronous such that the per capita growth rate is inversely proportional to the number of bradyzoites. While it may be surprising that the bradyzoites replicate in a way analogous to a factory producing a product, there may be factors such as nutrient availability, resource allocation, immune response, and other stress factors that may limit replication in cysts.

## Introduction

**Figure 1:**
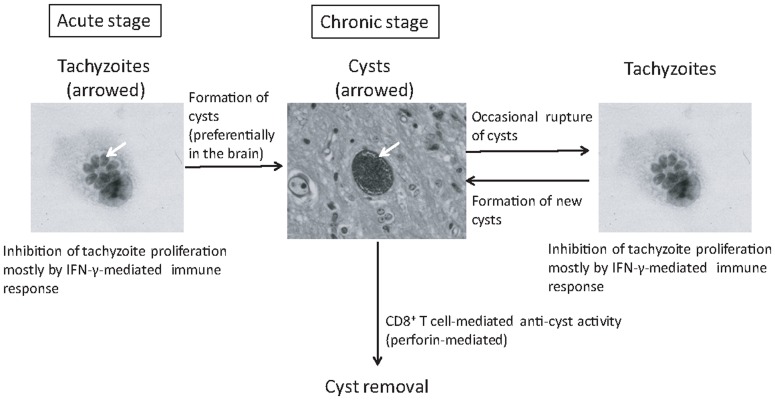
Schematic life cycle of *T. gondii*.

*Toxoplasma gondii*, an obligate intracellular protozoan parasite, is an important foodborne pathogen that can cause various diseases including lymphadenitis and congenital infection of the fetuses in humans. Infection occurs through ingestion of food or water contaminated with cysts or oocysts. The acute stage of infection is characterized by proliferation of tachyzoites in various nucleated cells. IFN-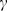-mediated immune responses limit tachyzoite proliferation [1]–[3] and the parasite establishes a chronic infection by forming cysts containing bradyzoites, primarily in the brain (Figure 1). Chronic infection with *T. gondii* is one of the most common parasitic infections in humans. It is estimated that 500 million to 2 billion people worldwide are infected with the parasite [4], [5].

During the chronic stage of infection, bradyzoites slowly replicate within the cysts and cyst sizes increase in response. In immunocompromised individuals such as those with AIDS and organ transplants, cysts can rupture resulting in release of bradyzoites, conversion of bradyzoites into tachyzoites, and proliferation of tachyzoites, which can cause life-threatening toxoplasmic encephalitis [6], [7] (Figure 1). Even in immunocompetent host, *T. gondii* cysts occasionally rupture during the chronic stage of infection [8]. In these cases, tachyzoite growth is controlled by the host's immune response, but the parasite is most likely able to form small numbers of new cysts (Figure 1). Such natural rupture of cysts and the formation of new cysts are thought to result in a wide range of *T. gondii* cyst sizes observed in the brains of chronically infected mice.

There is currently only limited information on the immune responses to the cyst stage of *T. gondii* [9], [10]. It was generally considered that *T. gondii* cysts cannot be recognized by the immune system. However, our recent study revealed that the 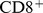 T cells have the capability to remove tissue cysts from the brains of infected mice [9]. Marked decreases in cyst numbers occur during the T cell-mediated anti-cyst immune responses, suggesting that the immunity-mediated removal of the cysts can prevent formation of new cysts (Figure 1). Therefore, host-pathogen interactions during the chronic stage of *T. gondii* infection appear to have two distinct processes. One is a natural rupture of tissue cysts that can result in formation of new cysts. The other is the T cell-mediated cyst removal not associated with formation of new cysts. In order to better understand the dynamics of host-pathogen interactions during chronic *T. gondii* infection, in the present study we developed a set of biologically based models of cyst growth and removal including both natural rupture and immunity-mediated removal of tissue cysts and compared these models with actual data on distribution of sizes of *T. gondii* cysts obtained from the brains of chronically infected mice.

## Methods

### Measurements of *T. gondii* Cysts Formed in the Brains of Mice

Previous studies by Hooshyar et al. [11] provided limited snapshots of the cyst size distributions in the brains of infected mice during the period of 2–4 months after infection. Typically, sizes of *T. gondii* cysts are viewed in terms of diameter. However, volume is biologically a more appropriate measure to indicate the size of cysts since it is expected to be proportional to the number of bradyzoites in a cyst. Hooshyar et al. assumed the shape of a cyst was ellipsoidal and measured the two diameters of the ellipsoid [11]. Based on their data, the mean volumes of cysts at 2, 3, and 4 months after infection were 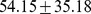 (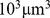), 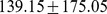 (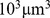), and 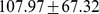 (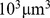), respectively. The number of cysts examined was 17 for each time point. There is a significant difference in the cyst volume between months 2 and 3 (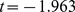, 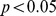), 2 and 4 (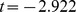, 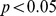), but not 3 and 4 (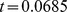, not significant). These studies support the assumption that cyst volume reaches a steady state distribution within 4 months after infection.

**Figure 2:**
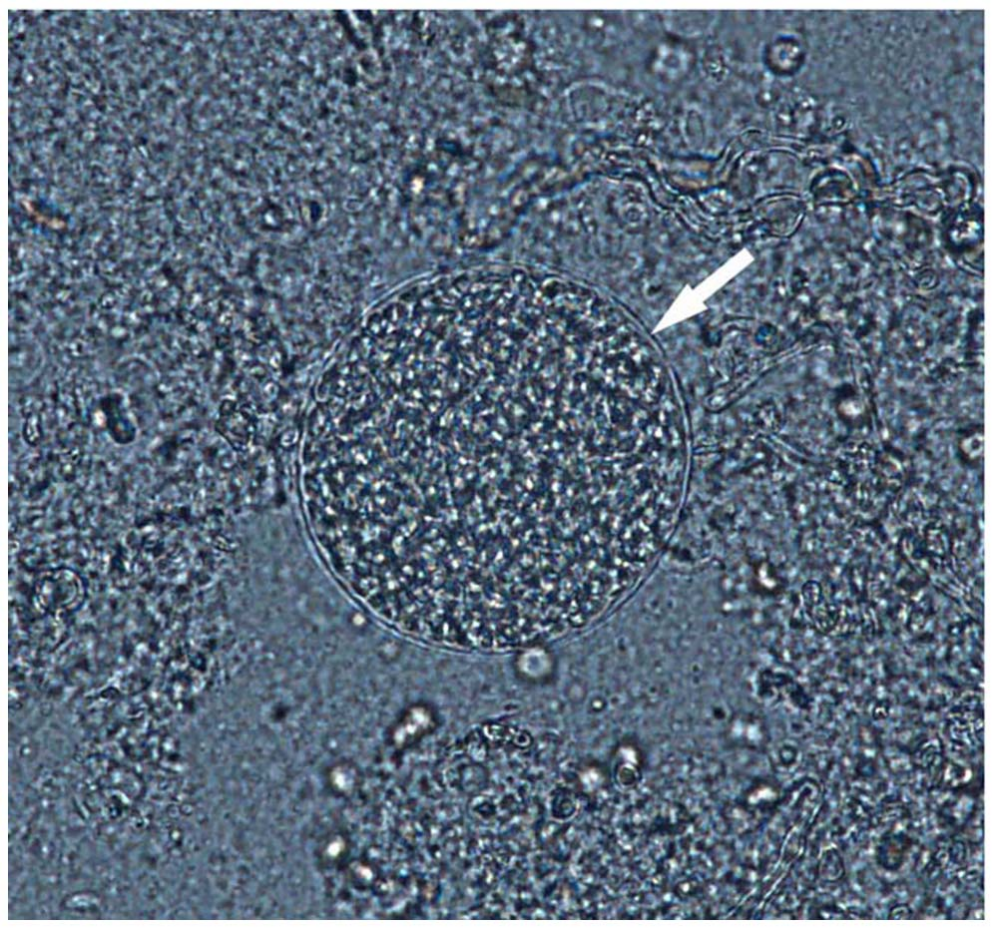
Example of photograph of a cyst from our experiment. Most of the cysts observed took on similar nearly circular cross section projections.

**Figure 3:**
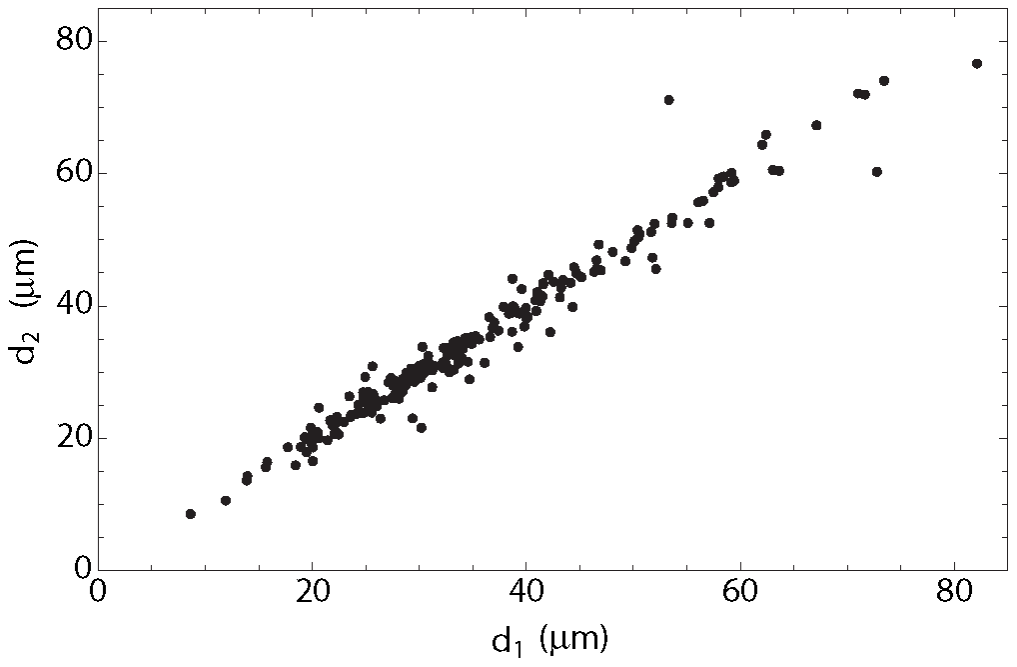
Relationship between the two measured diameters for each cyst. The effective diameter is computed as the geometric mean of the two measured diameters: 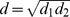.

In order to have a larger data set of cysts in the steady state during the chronic stage of infection, we measured sizes of over 200 cysts of *T. gondii* in the brains of mice at 6 months after infection. Female Swiss-Webster mice (Taconic, Germantown, NY) were infected intraperitoneally with 10 cysts of the ME49 strain (a type II strain) as previously described in [12]. *T. gondii* has three predominant clonal genotypes (types I, II, and III) [13]–[15]. Type II constitutes a majority of clinical cases of toxoplasmosis and asymptomatic infections in humans in North America and Europe [13], [15], [16]. Six months later, the brain of each of four mice was triturated in 1 ml of PBS [9]. Mouse care and experimental procedures were performed in accordance with established institutional guidance and approved protocols from the Institutional Animal Care and Use Committee. Four to six aliquots (20 microliters each) of each brain suspension were applied to microscopic examination using a Nikon Eclipse 90i microscope and a photograph was taken on each *T. gondii* cyst detected at ×400 magnification with a Nikon DS-Ri1 digital camera. Photographs of 50–56 cysts from each brain, a total of 213 cysts from four mice, were recorded (see Figure 2 for a photograph of a typical cyst). We measured the diameter of each cyst from two different angles using NIS Elements BR analysis 3.2 software (Figure 3; see also supplemental data). We calculated the volumes of each cyst using the two measured diameters by assuming an ellipsoidal shape: 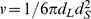, where 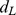 is the larger diameter and 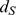 is the smaller diameter.

### Modeling Cyst Formation and Growth

**Figure 4:**
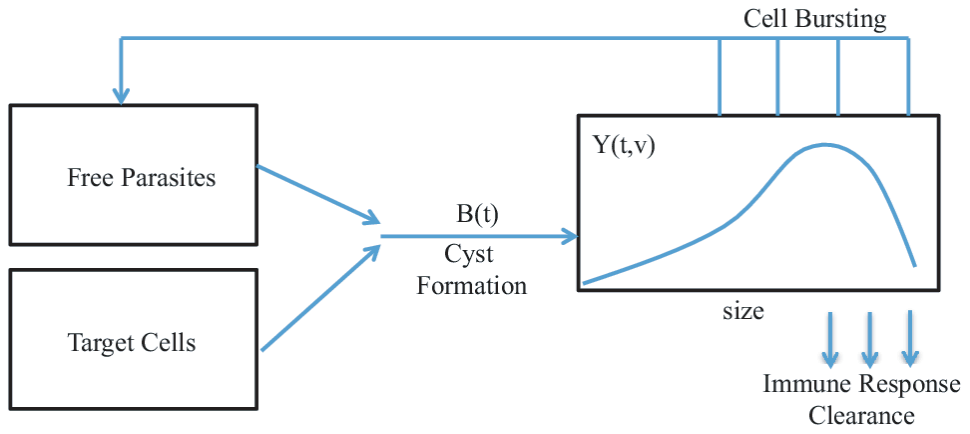
Chronic infection diagram of cyst-volume distribution model. Parasites infect healthy cells and begin replicating, causing the volume of the cyst to increase. The parasites burst at some rate and release new parasites into the system which are capable of infecting new healthy cells.

There have been several attempts to understand the biology of *Toxoplasma gondii* infection through mathematical modeling [17], [18], however, none of these previous efforts have tried to model the growth and distribution of cysts as a function of their volume. Because in this study we are solely interested in the distribution of cyst volumes, we do not explicitly model population of free bradyzoites, tachyzoites, and uninfected target cells and, instead, simply assume new cysts are being formed at some rate 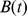. See Figure 4 for a schematic of the within-host system and Table 1 for definitions of the functions used in our model. Biologically 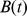 represents the rate at which uninfected target cells become infected by free parasites and begin forming intracellular cysts. Following [19], we model the growth of these cysts using a partial differential equation (PDE) structured by both time 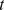 and cyst volume 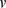. Specifically,
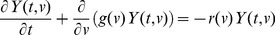
(1)where 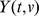 is the density of bradyzoite cysts of volume 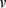 at time 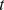, 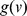 is the cyst growth rate, i.e. the rate at which the bradyzoite population grows within a cyst, and 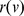 is the cyst removal rate, i.e. the sum of the rate at which encysted cells are either cleared by the immune response or through natural cyst bursting.

**Table 1:** Functions and their definitions.

Function	Biological Description
*B*(*t*)	The rate at which uninfected target cells are becoming infected at time *t*, leading to the formation of new cysts with volume *v*_0_.
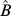	The rate at which uninfected target cells are becoming infected at steady state.
*g*(*v*)	Cyst volume growth rate. Equal to the rate at which bradyzoite population is increasing within a cyst.
*g′*(*v*)	First derivative of the cyst growth function *g*(*v*) with respect to *v*.
*r*(*v*)	Cyst removal via both immune response clearance and cyst bursting.
*v*_0_	Volume of newly formed cysts.
*v*_max_	Maximum possible cyst volume.
*Y*(*t*,*v*)	Absolute density of host cells infected with cysts of volume *v* at time *t*.
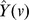	Absolute density of host cells infected with cysts of volume *v* at steady state.
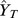	Total density of infected host cells and equal to 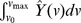 .
*y*(*v*)	Relative density of host cells infected with cysts of volume *v* at steady state and equal to 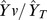

Conceptually, the PDE defined in Equation (1) describes how the density of cysts of size 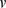 at time 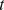 develops over time. For example, the first term on the left hand side of Equation (1) describes the ‘movement’ of cysts of size 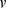 along the time variable 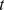. Since movement along the time axis is constant, we can think of the cysts as being carried along a conveyer belt along the 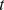 variable. The second term describes how growth ‘stretches’ or ‘compresses’ the distribution of 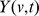 with cyst growth. For example, if we are considering the density of cysts in a region where 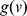 is increasing with 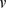, then the density of cysts 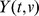 will be stretched out along the 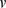 variable as larger cysts move more quickly along the axis. In contrast, if 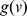 is decreasing with 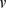 then 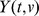 will be compressed along 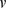 as smaller cysts ‘catch up’ with the larger cysts. Finally, if 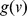 is constant with respect to 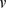, similar to with the time variable, the density of cysts 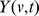 can be envisioned as moving along the 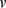 axis on conveyer belt. The removal term on the right hand side of Equation (1) describes the rate at which the cyst density 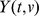 is being ‘siphoned off’ via the removal process. If the removal rate 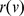 decreases/increases with 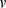, then larger cysts are removed at a lower/higher rate. If 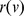 is constant with respect to 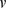, then the total density of cysts of a particular age (i.e. 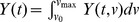) will decline exponentially with time 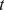.

Although these two cyst removal processes differ in that bursting can ultimately leads to the production of new cysts while immune response clearance does not, their effects on the relative distribution of cysts as a function of volume are indistinguishable and, hence, combined in Equation (1). Biologically, both 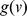 and 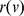 likely vary with the immune response state of the host. However, since we are focusing on the steady state of the system where the immune response state of the host is constant, we do not explicitly model this dependency. For simplicity, we assume that all new cysts have an initial volume 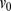. Based on our definition of 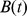 as the rate at which new cysts are formed, according to [20] the boundary condition for Equation (1) satisfies the equality,
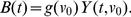
(2)


The general solution of Equation (1) can be obtained using the method of characteristics [21]. First, an inverse function must be determined to find the correspondence between size and time. Depending on a cysts initial volume, 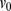, the current volume, 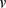, can be determined by some function that depends on the elapsed time since infection. This function, 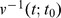 is the solution to 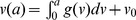, where 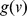 is growth rate. From equation (2), the equation for 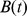 is the boundary condition. Then, the general solution is:
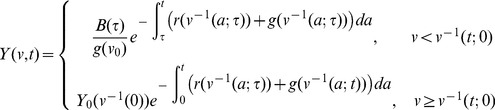
(3)where 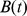 is the boundary condition (inflow of all new cysts into the system), 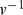 is the characteristic curve through the time-size domain that is defined by solving the inverse equation above, 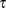 is the initial time we wish to model. See Calsina and Saldana for a complete derivation [21].

### Steady-State Solution

Although Equation (1) can be explicitly solved as a function of time (e.g. see [21]), here we focus solely on the steady state solution. Letting 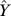 represent the steady state solution of Equation (1), that is, 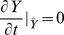. Under this condition, Equation (1) simplifies to the following ordinary differential equation
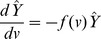
(4)where 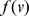 is a combined function of the cyst growth 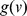 and removal 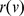 functions:
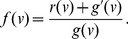
(5)Note that 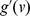 is the derivative of 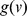 with respect to 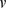. Equation (4) has a general solution of
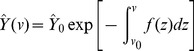
(6)where 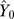 represents the steady state density of newly formed cysts and satisfies the boundary condition defined in equation (2) with 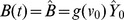.

Because the combined function 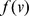 is a function of both 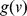 and 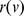 the first parameters of growth and removal functions, 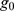 and 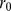 respectively, cannot be uniquely identified. Instead, they can be estimated only as ratios of one another, i.e. 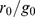, in this setting.

### Fitting Models to the Cyst Volume Estimates

Our data on cyst volume represents a random sample from the larger cyst population, in order to fit our models to this data we generate a probability density function 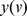 from our steady state solution. We investigate the steady state solution in Equation (6) under several different forms of growth and removal functions; see the function definitions in Table 2. We divide cyst density by the total cyst population size, 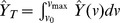 to get a probability density function for cyst size. Specifically,
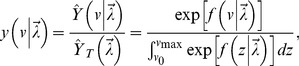
(7)where 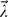 represents the parameters of a given combined function 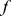 (e.g. 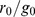 or 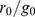 and 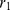). Using this probability density function, it follows that the negative log-likelihood 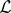 of a particular model and parameter set 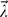 given a random sample of 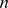 observed cyst volumes 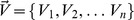 is simply,
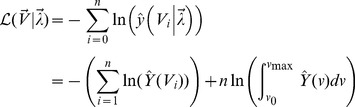
(8)


**Table 2:** Model selection results.

Index	Growth *g*(*v*)	Removal *r*(*v*)	AIC	ΔAIC	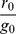	*r*_1_	*v*_max_
1	*g*_0_	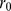	1915.88	0.33	0.03	N/A	N/A
2	*g*_0_	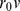	1990.32	74.77	0.0004	N/A	N/A
3	*g*_0_	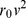	2064.06	148.51	10^−6^	N/A	N/A
4	*g*_0_	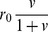	1916.21	0.66	0.03	N/A	N/A
5	*g*_0_	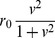	1915.55	0.0	0.03	N/A	N/A
6	*g*_0_	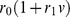	1917.88	2.33	0.0017	0.0	N/A
7	*g*_0_	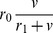	1917.87	2.32	0.03	0.07	N/A
8	*g*_0_	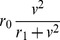	1917.12	1.57	0.03	8.74	N/A
9	*g*_0_ *v*	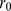	2812.87	897.32	0.07	N/A	N/A
10	*g*_0_ *v*	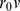	2487.93	572.38	0.002	N/A	N/A
11	*g*_0_ *v*	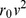	2467.12	551.56	2.2×10^−5^	N/A	N/A
12	*g*_0_ *v*	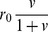	2670.9	755.35	0.13	N/A	N/A
13	*g*_0_ *v*	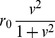	2670.1	754.55	0.13	N/A	N/A
14	*g*_0_ *v*	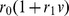	2489.93	574.38	8.07×10^−7^	2180.13	N/A
15	*g*_0_ *v*	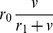	2489.93	574.38	2.70×10^6^	1.53×10^9^	N/A
16	*g*_0_ *v*	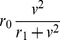	2469.12	553.56	3.01×10^6^	1.35×10^11^	N/A
17	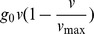	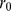	2794.98	879.43	0.045	N/A	1862.92
18	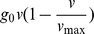	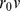	2487.92	572.36	0.0035	N/A	936.57
19	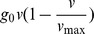	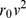	2436.17	520.62	0.000025	N/A	277.77
20	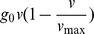	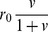	2687.36	771.81	0.082	N/A	2308.9
21	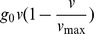	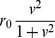	2749.31	833.76	0.045	N/A	1956.8
22	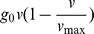	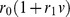	2460.84	545.28	0.000068	39.015	278.72
23	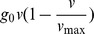	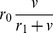	2460.51	544.95	3.74×10^6^	1.41×10^9^	277.77
24	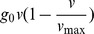	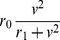	2510.48	594.93	0.60	1565.63	1138.84

The AIC is calculated using 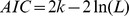, where 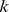 is the number of parameters and 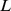 is the maximum likelihood for the model. Models 1–8 correspond to a constant growth function. Models 9–16 correspond to a linear growth function. Models 17–24 correspond to a logistic growth function. The removal functions in models 4, 12, and 20 are known as the one-parameter type II function. The removal functions in models 5, 13, and 21 are known as the one-parameter type III function. The removal functions in models 7, 15, and 23 are known as the two-parameter type II function. The removal functions in models 8, 16, and 24 are known as the two-parameter type III function. See Figure 6 for schematic representations of different growth and removal functions. For models 1–5 and models 9–13, 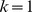 and the parameter is 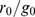. For models 6–8 and models 14–16, 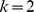 and the parameters are 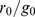 and 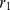. For models 17–21, 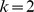 and the parameters are 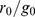 and 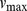. For models 22–24, 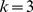 and the parameters are 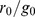, 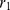, and 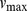.

For each model in Table 2 we estimated the corresponding model parameters 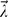 by minimizing 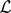 based on the observed data 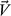 using the NMinimize routine in Mathematica 8.1. The minimal 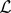 value and the total number of independent parameters were used to calculate the AIC value for each model. AIC and parameter estimates are also presented in Table 2.

## Results

**Figure 5:**
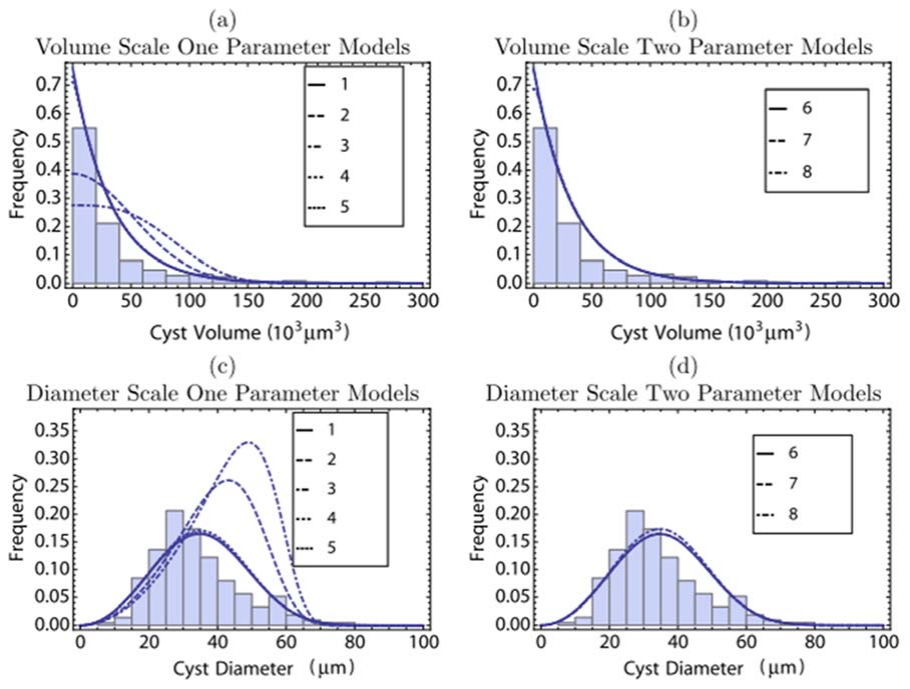
Comparison of probability distributions between experimental data and model selection results using the constant growth function: one-parameter models (indices 1–5) and two-parameter models (indices 6–8). Panels (a) and (b) show the distributions against volumes and panels (c) and (d) show the distributions against effective diameters. See Figure 3 for the definition of the effective diameter. See Table 2 and Figure 6 for definitions of different models.

We measured two diameters on each of 213 cysts detected in the brains of 4 mice at 6 months after infection in order to have a larger size of data on volume of cysts in the steady stage during the chronic stage of infection. Distributions of diameters measured and volume of cysts calculated from the diameters by assuming that cysts are in an ellipsoidal shape are shown in Figure 5. While the probability distribution on the diameter scale (Figures 5 (c) and (d)) is unimodal, the probability distribution on the volume scale (Figures 5 (a) and (b)) does not show modality. This difference is due to nonlinear transform between volume and diameter [22]. See the Methods section for calculation of the volume.

We developed a differential equation model to investigate the cumulative effects of unknown growth and removal functions on the cyst- size distribution. As a means for model selection, the Akaike information criterion (AIC) [23] was used to evaluate and compare different models; see Table 2. Based on information entropy, AIC is an estimate of the relative information lost for a given model. The AIC value of a model is calculated using its negative log-likelihood at the maximum-likelihood estimation (MLE) parameters and the number of parameters. Therefore, AIC provides a trade-off between a model's complexity and its goodness of fit. The 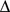
AIC of a given model is the difference between the lowest observed AIC value and the AIC value of the model [24].

**Figure 6:**
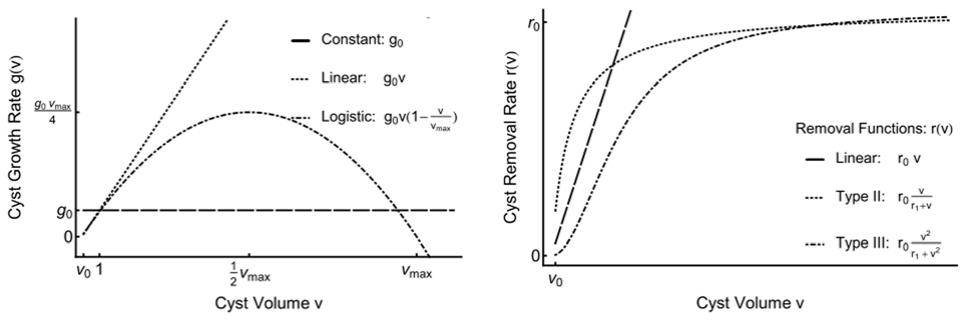
Schematic representations of the growth (left) and removal (right) functions.

We explored three different growth functions and eight different removal functions. A schematic illustrations of these functions are shown in Figure 6. More detailed descriptions of the function formalities can be seen in Holling [25]. To determine the cyst growth model that can fit best to the experimental data, we explore three different hypotheses as follows. The first hypothesis is that cysts grow at a constant rate i.e. 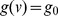, such that the cyst volume increases linearly with time (indices 1–8 in Table 2). Because bradyzoite number within a cyst increases with its size, this hypothesis corresponds to a per capita growth rate of bradyzoites that is inversely proportional to the number of bradyzoite, implying that bradyzoite replication is finely regulated and asynchronous within the cyst. The second hypothesis is that the cyst volume increases exponentially with time 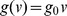 (indices 9–16 in Table 2). This corresponds to a constant per capita growth rate of within-cyst bradyzoites, implying that bradyzoites replicate independently of each other within the cyst. The third hypothesis is that the cyst volume grows logistically with time i.e. 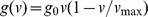 (indices 17–24 in Table 2). This hypothesis implies that bradyzoite replication is regulated within the cyst in a simple density dependent manner in which the per capita growth rate declines linearly with cyst volume. The AIC scores indicate that hypotheses two and three are not supported by the data. Therefore, we focused on various removal functions under hypothesis one. In regard to the cyst removal rate, models with constant (index 1), one-parameter type II (index 4), two-parameter linear (index 6), two-parameter type II (index 7), and two-parameter type III (index 8) functions all fell within 2.5 AIC units of the best model (index 5), which is a model with a one-parameter type III function. We can, however, clearly reject models where cyst removal rate increases linearly (index 2) or quadratically (index 3) in association with increases in cyst volume. Comparison between probability distributions of the experimental data and the models using constant growth function (indices 1–8) in Figure 5.

## Discussion

We have developed a mathematical framework to select the most appropriate mathematical descriptions for the growth and removal processes of *T. gondii* cysts through parameter fitting of experimental data obtained from the brains of chronically infected mice. Population growth often satisfies a linear or logistic growth function [26]. However, experimental data here supports a constant growth rate model, i.e., 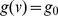. We calculated the volumes of cysts by assuming that cysts are in an ellipsoidal shape. We also performed the same analysis by assuming that cysts are in a spherical shape using the effective diameter (data not shown). In both cases, we reached the same conclusion. We assumed the cyst volume is proportional to the number of bradyzoites within the cyst. Therefore, a constant volume growth rate indicates that the number of parasites within the cyst increases linearly over time and the per capita growth of bradyzoites is inversely proportional to the number of parasites within a cyst. This probably suggests that bradyzoites do not replicate synchronously but each bradyzoite divide independently to produce a single new bradyzoite within a certain time interval. For example, a cyst may start with a given number of bradyzoites and a single new bradyzoite may be formed through replication every few hours. This is a contrast to tachyzoites of *T. gondii* or merozoites of malaria parasite. The tachyzoites and merozoites are the acute stage form of these parasites and they proliferate quickly after invading into host cells. On the other hand, tissue cysts of *T. gondii* are formed in the chronic stage of infection and the major purpose of cysts is most likely to persist within host cells, rather than proliferate. Therefore, it appears that tachyzoites and bradyzoites within cysts are under distinct regulatory mechanisms to control their proliferation. While it may be surprising that the bradyzoites replicate in a way analogous to a factory producing a product, there may be factors such as nutrient availability, immune response, and other stress factors that may limit their replication.

Based on the analyses on cyst growth described above, we performed parameter fitting of various removal functions with the constant growth rate. The best model was a one-parameter type III function; however several other removal functions performed similarly well and are indistinguishable from one another. Based on the AIC criteria, performances of the following functions (constant, type II, type III, and type III with two parameters) are indistinguishable for the constant growth rate model. Thus, the current data cannot distinguish between several removal functions. However, our analyses were able to reject models where cyst removal rate increases linearly or quadratically with increases in cyst volume. This result would suggest that removal of cysts is the outcome of a complex of multiple biological mechanisms.

In this study, we considered two removal processes: natural rupture and immune-mediated removal. Natural rupture of cysts may not occur simply based on the volume of cysts. It may also depend on cell-types of cyst-containing host cells and location of cysts in the brain. It has been shown that *T. gondii* can form cysts in both glial cells and neurons [27]–[29]. Removal of cysts by immune T cells and phagocytes could be independent of the sizes of the cysts contained in the infected host cells. To determine the specific removal function that fits best in experimental data, we would need to collect data on the transient dynamics and conduct corresponding studies. Moreover, the current study on steady state can only determine the ratio between the parameters 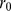 and 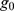. Transient data are also needed to estimate these parameters separately.

Recent studies suggested possible contributions of chronic infection with *T. gondii* with important diseases such as cryptogenic epilepsy and Alzheimer's disease [30], [31]. Thus, it is crucial to understand the mechanisms of host-pathogens interactions in the brain during the chronic stage of infection with this parasite for defining the pathogenesis of this common infection. The present study provided valuable information that may improve our understanding in this aspect. This study also demonstrated a power of mathematical modeling to provide the information that will be difficult to obtain directly from biological studies. In the present study, we obtained the data at only one time point of the chronic stage of infection. Having the data from multiple time points including the acute stage of infection and larger samples numbers at each time points will assist in understanding of dynamics of cyst growth and removal during the course of infection with *T. gondii*. These data will also assist in better understanding of the roles of natural rupture of cysts and immune response-mediated removal of cysts in the pathogenesis of cerebral infection with the parasite.

## Supporting Information

Dataset S1 Cysts from mouse 1.
(CSV)

Dataset S2 Cysts from mouse 2.
(CSV)

Dataset S3 Cysts from mouse 3.
(CSV)

Dataset S4 Cysts from mouse 4.
(CSV)
